# Use of Patient-Reported Outcome (PRO) Measures at Group and Patient Levels: Experiences From the Generic Integrated PRO System, WestChronic

**DOI:** 10.2196/ijmr.2885

**Published:** 2014-02-11

**Authors:** Niels Henrik Ingvar Hjollund, Louise Pape Larsen, Karin Biering, Soren Paaske Johnsen, Erik Riiskjær, Liv Marit Schougaard

**Affiliations:** ^1^WestChronicRegional Hospital West JutlandHerningDenmark; ^2^Department of Clinical EpidemiologyAarhus University HospitalAarhusDenmark; ^3^Department of Occupational MedicineRegional Hospital West JutlandHerningDenmark; ^4^Public Health and Quality ImprovementCentral Denmark RegionAarhusDenmark

**Keywords:** data collection, decision support systems, health care economics and organizations, health education, Internet, longitudinal studies, outcome assessment, patient-reported outcomes, questionnaires, quality improvement

## Abstract

**Background:**

Patient-reported outcome (PRO) measures may be used at a group level for research and quality improvement and at the individual patient level to support clinical decision making and ensure efficient use of resources. The challenges involved in implementing PRO measures are mostly the same regardless of aims and diagnostic groups and include logistic feasibility, high response rates, robustness, and ability to adapt to the needs of patient groups and settings. If generic PRO systems can adapt to specific needs, advanced technology can be shared between medical specialties and for different aims.

**Objective:**

We describe methodological, organizational, and practical experiences with a generic PRO system, WestChronic, which is in use among a range of diagnostic groups and for a range of purposes.

**Methods:**

The WestChronic system supports PRO data collection, with integration of Web and paper PRO questionnaires (mixed-mode) and automated procedures that enable adherence to implementation-specific schedules for the collection of PRO. For analysis, we divided functionalities into four elements: basic PRO data collection and logistics, PRO-based clinical decision support, PRO-based automated decision algorithms, and other forms of communication. While the first element is ubiquitous, the others are optional and only applicable at a patient level. Methodological and organizational experiences were described according to each element.

**Results:**

WestChronic has, to date, been implemented in 22 PRO projects within 18 diagnostic groups, including cardiology, neurology, rheumatology, nephrology, orthopedic surgery, gynecology, oncology, and psychiatry. The aims of the individual projects included epidemiological research, quality improvement, hospital evaluation, clinical decision support, efficient use of outpatient clinic resources, and screening for side effects and comorbidity. In total 30,174 patients have been included, and 59,232 PRO assessments have been collected using 92 different PRO questionnaires. Response rates of up to 93% were achieved for first-round questionnaires and up to 99% during follow-up. For 6 diagnostic groups, PRO data were displayed graphically to the clinician to facilitate flagging of important symptoms and decision support, and in 5 diagnostic groups PRO data were used for automatic algorithm-based decisions.

**Conclusions:**

WestChronic has allowed the implementation of all proposed protocol for data collection and processing. The system has achieved high response rates, and longitudinal attrition is limited. The relevance of the questions, the mixed-mode principle, and automated procedures has contributed to the high response rates. Furthermore, development and implementation of a number of approaches and methods for clinical use of PRO has been possible without challenging the generic property. Generic multipurpose PRO systems may enable sharing of automated and efficient logistics, optimal response rates, and other advanced options for PRO data collection and processing, while still allowing adaptation to specific aims and patient groups.

## Introduction

The US Food and Drug Administration defines patient-reported outcome (PRO) as a measurement based on “any report of the status of a patient’s health condition that comes directly from the patient, without interpretation of the patient’s response by a clinician or anyone else” [1]. This definition emphasizes a generic and patient-oriented perspective, but also a systematic aspect. From the time of Hippocrates, information originating from the patient has been considered indispensable, and still today, few diagnoses can be established and few treatments be monitored without information from the patients. However, information from the patient is normally interpreted and reported by a clinician [2], and consequently this information is not in the form of a PRO.

PRO was initially developed for research, and with the introduction of the term health-related quality of life, systematic measurement of PRO was adopted for research in a number of clinical specialties [3]. During the last decades, PRO has been identified as a tool for hospital performance assessment [4], and recent initiatives include the United Kingdom’s policy to encourage and request use of PRO for assessment [5,6] and the nationwide use of PRO to compare Medicare health plans in the United States [7]. In Sweden and Denmark, nationwide use of PRO was initially driven by the medical profession’s focusing on improving clinical care, and PRO was introduced in some disease-specific national clinical registers in 2000 [8,9].

The evolution of PRO is now tending to return to its origin: the interaction between the patient and the clinician in daily clinical practice. The applications of PRO in clinical practice, include screening tools, monitoring tools, decision aids, and as a means of monitoring the quality of patient care [10-12]. Reviews find evidence of improved patient care, patient-clinician communication, and better identification of treatment symptoms and psychosocial problems, while findings with respect to an effect on subsequent patient outcomes are less consistent [10,12-15]. In a recent comprehensive review of randomized trials, it was concluded that PRO used for consultation support provides patient-centered care, ensuring that patient-reported symptoms guides the clinical decisions [15], and it has been found that PRO and clinical judgments produce complementary data, which, when combined, provide a more accurate description of the patients’ symptoms [14]. PRO data collected for individual patients may also be aggregated and used at a group level for research and to compare quality of care across providers [11,16].

Challenges to the use of PRO vary according to the specific aims, but high response rates are almost always warranted. At the group level (research and performance assessment), estimates based on low response rates are prone to selection bias. At the individual level (eg, in PRO-based outpatient clinics), low participation rates undermine the usefulness of any clinical PRO application. If a PRO assessment is to be completed while the patient is physically present in the hospital outpatient clinic, patient kiosks may be used to collect PRO data electronically (ePRO) [17]. In these systems, patients are required to fill out forms before a scheduled appointment, and high response rates may be obtained with some gentle prompting from the hospital staff. However, when the aim of a PRO assessment is to evaluate the need for a hospital visit, the PRO assessment must be obtained while the patient is still at home (TelePRO). The same is the case in most epidemiological and quality assurance projects. A number of PRO systems have introduced Web-based questionnaires in which the data could be used for such purposes, and there have been high expectations of easy and costless Web-based data collection, once the vast majority of the population is online. Unfortunately, it is evident from randomized studies that data collection that relies only on Web-based questionnaires filled in at home reaches response rates of only 20% to 45% [4,18-20], while combined with paper-based methods (mixed-mode) may reach 75% or more dependent on the number of reminders [20]. Reports of high rates in Web-based systems are generally from populations selected on actual Web use (eg, studies in which Web use is a prerequisite for enrollment) [21]. As a consequence, TelePRO data collection can seldom rely only on a Web-based solution. However, solutions that apply paper questionnaires are normally considered to increase the logistic challenges considerably, and may delay the timely availability of the data for the clinicians.

The response is highly dependent on successful logistics, and adherence to a proper protocol (eg, nonrespondents reminded as scheduled) is crucial to obtain a high response rate and low attrition. Questionnaire logistic often receives little scientific attention and may even be considered a trivial technical issue. Even though logistic and scientific challenges are similar across diagnostic groups and applications, most PRO systems have been applied to a single-patient group [22]. By development of generic (not diagnosis-specific) systems, methodological and economical large-scale benefits may be achieved, and, moreover, new possibilities for PRO-based research across traditional medical specialties may emerge [22].

The aim of the present paper was to describe methodological and organizational experiences with a generic PRO system, WestChronic, which has been used to collect PRO data among a range of diagnostic groups and for a range of purposes.

## Methods

### Overview

This paper reports and discusses all projects implemented in the PRO system WestChronic. We found no appropriate analytical frameworks in the literature, and the classification was defined as post-hoc, based on actual experiences.

### The WestChronic System

The first version of the generic PRO system, WestChronic, was developed in 2004 by the first author for mixed-mode (Web and paper) collection of PRO data for research purposes in clinical epidemiological studies with repetitive measurements. Due to feasibility and high response rates, it was decided to develop this system into a flexible, multipurpose PRO system intended to facilitate adaption to the projects needs instead of requesting projects to adapt to a system. WestChronic supports dynamic mixed-mode data collection [23] with Web or paper forms as well as communication to the patient and clinician with personalized postal letters, emails, and text messages. All information regarding implemented projects, items and questionnaires, communication, users, and patients resides in tables in a Structured Query Language database, and all administration of projects, questionnaires, users, and patients is supported by the server software and managed in browser windows. The system automatically encourages patients to adopt the Web method and to be approached by email. A description of the dynamic mode-switching algorithms can be found in Multimedia Appendix 1. All Danish citizens are assigned a unique 10-digit central personal registry (CPR) number, and constantly updated information on current postal address and vital status is available from the national CPR registry. This information is automatically collected online prior to any approach to patients. On-demand printing of questionnaires and letters as well as scanning of incoming questionnaires and subsequent optical character recognition is controlled by the server software, and results with regard to all variables end up in result tables for the individual implemented projects in the same database, irrespective of whether Web or paper forms are used and are instantaneously accessible. All data transactions fulfill conditions established by the Danish Data Protection Agency. WestChronic may implement an arbitrary number of PRO projects with individual questionnaires, protocols, patients, and users. For the patient and clinician, each implemented project appears as a unique PRO project with its own logo, domain, website, email address, accompanying letters, contact information, etc. At present WestChronic includes 1756 items in 92 questionnaires, and 158 templates for personalized letters and emails.

### Data Collection and Analysis

#### Overview

Data from all implemented projects were collected according to routine by the WestChronic system. Response rates were calculated as the minimal response rate (RR1) [23].

**Figure 1:**
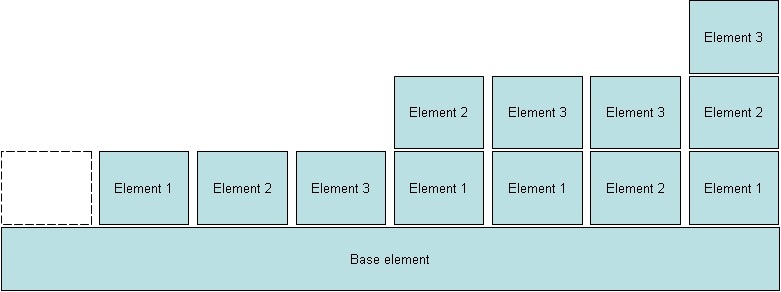
Possible combinations of the base element and three optional elements involved in clinical use of PRO. Base: PRO data collection and logistic, Element 1: PRO-based overview for clinical decision support, Element 2: PRO-based automated decision algorithms, Element 3: Other forms of communication.

We divided the function of the PRO system into a number of elements, the first of which is ubiquitous in any PRO application, while the other three are optional (Table 1 and Figure 1).

PRO data may be collected in the outpatient clinic or at a distance (eg, from home). We will refer to the latter as TelePRO. The mode to record the PRO data may be based on paper forms or electronic devices. Although paper modes may involve sophisticated electronic procedures like on-demand printing and optical character recognition, we will restrict the term ePRO to Web-based interfaces, tablet computers, other hand-held devices, and interactive voice response [17].

**Table 1:** Elements of clinical application of patient-reported outcomes (PRO).

Element		Content
Base element	PRO data collection and logistics	Questionnaire (items)
		Criteria for inclusion and termination
		Data collection modes: Web, paper, interview
		Approach modes: letter, email, telephone, texting
		Schedules of questionnaires/reminders
Optional element 1	PRO overview for clinical decision support	Categorization of PRO for clinical decision support
		Course-oriented graphic overview
Optional element 2	PRO-based automated decision algorithms	Decision tree
		Action protocol
Optional element 3	Other forms of communication	Two-way communication
		One-to-many communication

#### PRO Data Collection and Logistic (Base Element)

The base element is mandatory for any application of PRO, and may range from a photocopied paper form handed out to patients on arrival, filled in and used as is in the subsequent visit, to advanced computerized systems handling all processes. A PRO system may implement and manage some or all relevant elements of a protocol and may also support the logistics for the collection of PRO data. Crucial issues include the definition of content and development of the actual PRO questionnaire: the validity, reliability, acceptability to patients and clinicians, and the relevance with respect to the purpose of the collection of PRO data [24,25]. Other issues include the process of implementation, pilot-tests, and methods to collect and integrate user feedback and experiences. Issues of importance to response rate and usability, include the offered modalities (Web-based, paper-based), the offered options, and level of automation of the protocol (administration of implementations, items, handling of subjects, reminders, data import and export).

#### Optional Element 1: PRO Overview for Clinical Decision Support

A PRO system may enable the clinician to access and overview systematically collected PRO data on symptoms, functional status, and health-related quality of life that can support symptom monitoring, consultation support, and clinical decision-making [11,26]. The results of PRO assessments may be used longitudinally to monitor the course of symptoms and to flag symptoms that need further attention during an outpatient visit. The procedure may range from using the paper form as is as a checklist during the interview, to using graphical display systems fully integrated within an electronic health record (EHR) system, in which an overview is presented to the clinician, who can use it for clinical decisions together with other available clinical and laboratory data. It is crucial that the items used for decision support are relevant for the situation seen from the point of view of the patient as well as the clinician.

#### Optional Element 2: PRO-Based Automated Decision Algorithms

A PRO system may be designed to make automatic decisions. As a screening tool, PRO assessments may be used to identify patients that need attention as well as patients that do not need attention at the moment. The design may range from a simple score calculated by hand by the clinician and compared with published cut-off values, to automated computer algorithms that include actual absolute scores and intra-individual changes with respect to previous scores. Crucial issues include the risk of false-positive and false-negative results of the algorithm. In statistical terms this is expressed by sensitivity, specificity, and predictive values. Furthermore, the algorithm should be acceptable and meaningful for both patient and clinician.

#### Optional Element 3: Other Forms of Communication

Normally, researchers or clinicians define the content of the PRO questionnaire, prompt the patient to answer, and collect the data, but some functions in a PRO system may go beyond the one-way, one-to-one flow of information. It is noteworthy that the definition of PRO does not impose strict demands on the origin of a PRO assessment or prerequisites regarding who initiates the communication.

Figure 1 displays the eight possible combinations of the base element and the three optional elements.

## Results

### Summary

Overall, the WestChronic system has so far implemented 22 PRO projects within 18 diagnostic groups. By January 2014, a total number of 59,232 questionnaires have been collected from 30,174 patients. The characteristics of all the PRO projects are presented in Tables 2 and 3 by primary aim in order of increasing logistic and organizational complexity.

**Table 2:** Characteristics of 22 projects involving implementations of a generic PRO system. Projects with group level use (n=8).

	A: Clinical epidemiological research	B: PRO for clinical databases	C: PRO monitoring for administrative purposes
Level of aggregation	Group	Group	Group
Implemented projects	3	4	1
Invoked elements (Figure 1)	Base	Base	Base
Patients	Breast cancer IHD Stroke	Prostatic cancer Renal cancer Esophageal cancer Lung cancer	Stroke
Recruitment	Hospital registers/clinical databases	Clinical databases	Hospital registers
Primary aim	Research	Hospital performance assessment	Hospital performance assessment
Extension	Regional	National	Regional
In operation from	2004	2011	2012
Patients (Jan 2014)	11,898	8278	2735
Questionnaires/ patient	2-23	1-2	3
Response rate (primary)	81%-85%	93%	78%
Response rate follow-up	91%-99%	N/A^a^	96%

^a^Not applicable

**Table 3:** Characteristics of 22 projects involving implementations of a generic PRO system. Projects with patient level use (n=14).

	D: PRO for clinical overview (AmbuFlex I)	E: PRO for automated cancelling of visits	F: PRO for screening	G: PRO for clinical decision support (AmbuFlex II)	H: Other forms of communication
Level of aggregation	Patient	Group/patient	Group/patient	Patient	Patient
Implemented projects	5	3	2	3	1
Invoked elements (Figure 1)	Base+element 1	Base+element 2	Base+element 2	Base+element 1, 2	Base+element 1, 3
Patients	Chronic heart failure Rheumatoid arthritis Renal failure Lung cancer Prostatic cancer	Hip/knee replacement Endometriosis	Acute Coronary Syndrome Heart transplant	Epilepsy Sleep disorders Neuromuscular diseases	ADHD^a^
Recruitment	Preadmission assessment	Clinic referral	Hospital registers/clinical referral	Clinic referral	Clinic referral
Primary aim	Clinical decision support	Efficient use of resources	Screening for depression	Clinical decision support	Communication (therapists and patient)
Extension	Local	National, selected hospitals	Local	Regional	Local
In operation from	2009	2011	2011	2012	2012
Patients (Jan 2014)	741	1639	1740	3120	23
Questionnaires/ patient	No limit	3	1/no limit	No limit	No limit
Response rate (primary)	75%	N/A	88%	93%	N/A
Response rate follow-up	82%	97%	N/A	99%	N/A

^a^Attention deficit hyperactivity disorder

### PRO Data Collection and Logistic (Base Element)

#### Overview

PRO data were collected in mixed-mode with paper- and Web-based questionnaires in all projects except three, where only the Web-based method was applied. Three reminders were applied in project type G and two in all other, except in project type A/stroke, where no reminders were applied.

#### Participation Rates and Attrition

The participation rates for the implemented projects are displayed in Table 2. In all projects, the patient was clearly informed that participation was voluntary. The range in response rates to the first PRO questionnaires was between 75% and 93% (median value 85%). The highest rates (93%) were found in project type G/epilepsy (clinical decision support, n=2882 patients) and project type B/prostatic cancer patients (hospital assessment, n=7423), while the lowest (77%) were found among stroke patients (epidemiological research, n=3575). During follow-up, the rates were between 82% and 99% (median value 96%). Due to different protocols (eg, number of reminders varied between zero and three), the rates are not directly comparable.

In project types A, B, and C (Table 2) PRO data from 8 different diagnostic groups were collected for use at a group level, and therefore none of the three optional elements were in use. Experiences related to the content of the PRO questionnaires for these projects will be described here.

In project type A, the three projects, include patients with breast cancer, ischemic heart disease, and stroke. Patients are monitored with PRO by multiple measurements over a span of 2 to 6 years. The aim is to describe prognosis using PRO data regarding symptoms and functioning, and to analyze PRO variables as risk factors for medical and social outcomes [20,27-30].

In project type B, PRO data are collected nationwide for patients with four malignant diseases: prostatic cancer, renal cancer, esophageal cancer, and lung cancer. The aim is to include PRO measures in existing national clinical registers used for research and hospital performance assessment.

In project type C, in an ongoing reorganization of the treatment of stroke in the Central Region of Denmark, it was decided to collect PRO data consecutively in all patients to monitor possible effects.

#### Development of PRO for Use at the Group Level

PRO data collected for use at the group level (clinical epidemiological research and hospital performance assessments) must comply with the usual demands regarding validity and reliability [1], and these issues will not be described further here. Due to the generic property of WestChronic, valid scales can be applied across projects, which make it easy to implement new projects using scales already in the bank simply by selecting them from drop-down menus. In such cases, new projects may be implemented very quickly.

Experiences related to the content of PRO for projects with applications at a patient level will be described below in connection with the corresponding optional element.

### PRO Overview for Clinical Decision Support (Optional Element 1)

#### Overview

**Figure 2:**
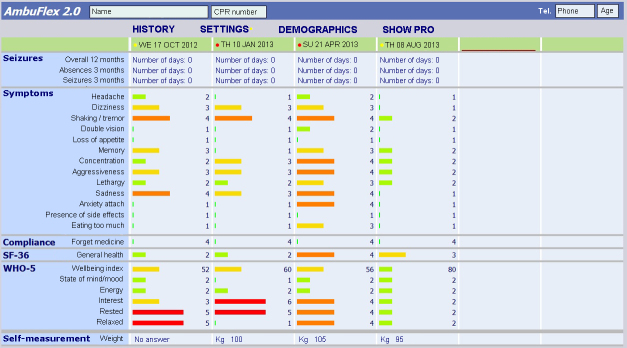
Screenshot captured from the PRO-based overview accessed from the EHR of the Central Denmark Region. The color codes in the upper row indicate the result of the automated PRO algorithm (red: definite need of contact; yellow: possible need of contact; green: no need of contact). Labels were translated from Danish.

In projects aiming at clinical decision support, the core element is a graphical overview over the course of PRO. The clinician is presented with a graphical view of the course of selected PRO variables displayed within an EHR in the same context as the clinical data. A screen shot capturing the AmbuFlex II implementation is shown in Figure 2. Each column represents a PRO assessment. Color codes signal the severity of the symptom. The actual wording of the question as well as the answer is displayed as a “pop-up tip” when the user puts the mouse icon over the displayed bar. Vertically, the overview presents the actual situation and horizontally the PRO course over time with regard to symptoms, functional level, etc.

The PRO overview is used in two different situations: (1) in telePRO to evaluate and decide whether the patient needs a visit, and (2) as consultation support to identify and flag important symptoms that need focus and attention at an outpatient visit or in a telephone consultation.

#### Facilitation of Efficient Visits in Outpatients With Heart Failure (AmbuFlex I: Project Type D)

Patients with chronic heart failure often need treatment with multiple pharmacological substances. During the period in which patients are seen in the outpatient clinic, medical therapy is up-titrated, and patients are scheduled for frequent visits to monitor treatment results, identify side effects, and ensure compliance. PRO questionnaires were filled out by the patient before the visit to facilitate more efficient visits by flagging important symptoms. Furthermore, the overview is used for telephone consultations, enabling these to be shorter and more comprehensive [31]. The application as a screening tool before a telephone consultation was not a specific aim, but turned out to be the most significant issue with respect to both quality and time saving in PRO projects using a graphical PRO overview. The same method is used in patients with epilepsy, rheumatoid arthritis, renal failure, sleep disorders, neuromuscular diseases, lung and prostatic cancer, and endometriosis (Project type D+G).

#### Decision Support in Outpatients With Epilepsy (AmbuFlex II, Project Type G)

Patients with epilepsy are normally followed-up as outpatients at a neurological clinic, usually with 1 to 4 appointments yearly. PRO questionnaires are used to evaluate whether the patient needs a visit or not. If not, the patient automatically receives a new PRO questionnaire after a preset interval (eg, 3 months). The procedure consists of two steps: an automated decision in patients with obvious clinical problems and patients with no obvious problems at the moment (optional element 2) and a PRO-based clinical decision support in the remaining patients. Overall, for 48.75% of the PRO questionnaires no additional contact to the patient was needed, while the remaining 51.25% had a subsequent follow-up visit or a telephone consultation. The same method is used in patients with sleep disorders, neuromuscular diseases, and prostatic cancer.

#### Development of PRO for Clinical Overview

The PRO-based overviews use a PRO assessment to reflect clinical aspects as they are met in the daily clinical practice for that particular group of patients. The clinician, who makes the decision based on the PRO overview, still has the professional responsibility in case of an erroneous decision. Our experience is that it is vital that clinicians have full confidence in the system, even at the item level (face validity). The content of the PRO is negotiated based on iterative inputs from clinicians, review of the literature, and anthropological interviews with patients [25,32]. For new items constructed in this way, identical 5-point Likert scales are used to assess severity and frequency of symptoms. Pilot tests and semistructured interviews before implementation are used to identify problems such as relevance of items, clarity of wording, ambiguity of items, and lack of important issues. After a pilot test, the PRO application is put in operation, and a parallel iterative process is launched in which experiences are continuously evaluated, and items and mode of display revised as a running process until saturation is reached after 2 to 4 months. We have experienced that such projects are easily transferred to outpatient clinics for the same patient group without any modification, even though the clinicians are invited to suggest such. Thus, an implementation seems to be specific for a patient group, not for a location. The implementations have been evaluated from a clinical as well as a patient perspective with positive conclusions [33].

### PRO-Based Automated Decision Algorithms (Optional Element 2)

#### Overview

An automated PRO algorithm was applied in project types E, F, and G. As a part of the implementation process, an algorithm for each specific group of patients was developed and programmed into the server software.

#### Screening for Depression and Anxiety (Project F)

According to Danish clinical guidelines, all patients discharged from hospital after an ischemic heart attack should be screened for depression and anxiety 6 weeks after admission. Due to logistic challenges, this is rarely accomplished. The patients are recruited consecutively from hospital discharge registers, and 6 weeks after admission they are mailed a generic questionnaire on depression and anxiety [34]. An automated algorithm based on published cut-off values divide patients into nine groups according to no, moderate, or severe symptoms on the two scales. Based on these values, WestChronic automatically generates a personalized letter with the results of the screening, and if moderate or severe symptoms are present, the patient is advised to consult a general practitioner and bring along the letter. The same method is now extended to heart transplanted patients.

#### Automated Canceling of Postoperative Follow-Up Visits (Project Type E)

According to guidelines, patients with hip and knee replacements are invited to a follow-up visit 3 months after surgery. Several studies have documented that few of these visits have any clinical consequences and could be cancelled if satisfactory information on, for example, pain and difficulty in walking were available [35]. Patients were included at the preoperative examination in selected hospitals in 4 of 5 Danish regions. The automated algorithm was based on published values from well-established disease-specific questionnaires on symptoms and functioning [36,37]. At the beginning of the questionnaire we included an additional item: “You may wish to have an outpatient clinic visit regardless of these clinical factors and you can indicate your preference here”. This option was ticked off by 291 patients (27.3%). If scores were below thresholds and the patient did not indicated an absolute wish for a follow-up visit, the department was electronically informed that the scheduled follow-up visit should be cancelled. The same method is used in patients with endometriosis, where the algorithm is based solely on the patient’s wish of a visit.

#### Automated Handling of Patients that Clearly Need Attention and Those that Do Not Prior to Clinical Decision Support (Project Type G)

Clinical decision support among outpatients with epilepsy is described above. First, however, patients obviously needing attention and those that do not are handled automatically. Based on the incoming PRO data, the server algorithms simultaneously categorize the patients’ present condition into red, yellow, or green status (red status: the patient should be seen or contacted; green status: no action is needed). In the latter case, clinicians are not notified or involved at all, and at the scheduled time (eg, 3 months) a new questionnaire is automatically printed out and mailed or emailed to the patient. The PRO assessments that could not be processed automatically are assigned yellow status, meaning that a clinician shall inspect the PRO overview (decision support; optional element 1). Examples of inducers of red status are self-reported aggravation of seizures or planning of pregnancy. We allow the patient to overrule the automated decision with the same question as mentioned above. Nonresponders and patients who indicate they want a personal clinical contact are categorized to red status. WestChronic keeps track of patients with red and yellow status, and, if no action is taken by a clinician before a deadline, the server software reacts with reminders to the clinician on duty and, if ignored, alarms the system supervisors. Among 2766 questionnaires (November 2013) 37.8% were handled automatically (10.3% green and 27.5% red). Among patients with epilepsy, 27.3% indicated an absolute wish for a clinical contact.

#### Development of PRO for Automated Decisions

For simple screening purposes with defined binary outcomes (depression, inadequate function after surgery) existing PRO scales with documented sensitivity and predictive values were used. In more complex clinical decisions in which the whole situation of a patient needs to be evaluated, in several cases no PRO instrument was available or applicable. In these applications the goal is to have a false negative rate of zero, whereas the rate of false positive is of less concern. When PRO is intended for automated decisions, both content (items) and threshold need to be defined and documented with respect to sensitivity and specificity. Even if an obvious candidate for the PRO questionnaire exists, it is only possible to extract a cut-off value from the literature if the aims are identical. This was the case in screening for depression and canceling of postoperative visits. In the other projects, we had no predefined cut-offs to rely on. Initially, we gave priority to sensitivity in order to identify all cases that would be identified in a normal practice. When experience was gathered, cut-offs were adjusted by consensus conferences.

### Other Forms of Communication (Optional Element 3)

#### Overview

In some projects there was need for information beyond that provided by predefined PRO questionnaires delivered from the patient to one or more clinicians.

In the very first PRO project (breast cancer, project type A), we gave the patient the possibility to log on and review her own course of symptoms over time. However, to comply with the demands from the Danish Data Protection Agency, we had to apply rather complicated procedures. A shared secure log-on procedure has now been provided at the national health website from which the patient can obtain a link to the personal site at WestChronic. In future implementations, the patient will be able to see an overview similar to what is presented to the clinician (optional element 1).

#### Communication and Shared Knowledge in Patients With Attention Deficit Hyperactivity Disorder (ADHD)

The treatment of patients with severe ADHD may involve several therapists and social workers (project H). The project attempts to promote an overview of the situation among a group of complex patients with often quick shifts in condition and surroundings, and where continually shared updated knowledge is crucial for all partners, including the patient and relatives. The system includes PRO as well as a Web-based communication area in which structured as well as unstructured information can be shared between all parties, including the patient. The psychiatric department creates a record for new patients and decides which partners are relevant in each particular case. Partners are labeled according to their role (eg, patient, community psychiatric nurse, municipal social worker, outpatient clinic nurse, relative). Each patient is assigned 1 main contact person at a time. At the beginning of treatment, this would typically be a member of the psychiatric team. Any partner, including the patient, can create a new communication. The communication element is accessed from the PRO-based graphical overview for the particular patient. The patient participates in the following ways: first, s/he fills in the PRO questionnaire, which is graphically displayed at the initial page where all partners enter the system. Second, s/he has access to exactly the same written information as all other partners. Finally, s/he may create a communication to all partners. The psychiatric team accesses the system through the EHR system, while other professional partners obtain accesses after logon to their local area network. The patient obtains access via secure login at the national health website.

## Discussion

### Principal Findings

The generic PRO system WestChronic has so far enabled implementation of 22 PRO projects. It has been possible to develop and integrate all proposed protocols for data collection and processing. The system has achieved high response rates, and the attrition in longitudinal projects has been limited. We presume that the relevance of the PRO, the mixed-mode principle with integration of Web and paper PRO together with automated procedures that enable strict adherence to the schedules of reminders, has contributed to the high response rates. Furthermore, it has been possible to develop a number of approaches and methods for the clinical use of PRO without challenging the generic nature of the system.

Several articles dealing with features of and experiences with PRO systems have been published [11,14,15,22,26,38-40], but we are aware that other systems exist that have not been reported in the scientific literature. This reflects the notion that PRO systems are often considered a simple product rather than a key feature of clinical or scientific practice. As a consequence, a number of systems have been developed and promoted for specific aims and patient groups, and the potentials for sharing of expensive technologies and large-scale advantages are lost [22]. A fully equipped PRO system with maximal automation of procedures is technologically advanced, albeit essentially generic in nature. Any aim regarding the collection of PRO measures (epidemiological and clinical research, quality assurance, hospital performance assessments, and clinical use at the patient level) may benefit from systems capable of easy implementation, connection to external databases, and high performance with respect to response rates no matter for what purpose. As an example, in WestChronic 83 programmed scripts meet the needs of all functions, including the integration of Web and paper forms. Only two of these scripts are dedicated to specific projects (project type D+G). We believe that we can benefit from these generic features and still allow patients and clinicians to adapt to the specific demands and wishes in the individual projects.

The potential benefits of PRO measures in clinical practice have been described with respect to improvement of quality of care, better symptom assessment, more patient-centered care, and more efficient use of resources [4,12,26,41]. However, as pointed out by Donaldson et al [42], implementation of PRO as an adjunct and added task to usual care will not ensure that these aims are fulfilled. The potential emerges when PRO measures are fully incorporated into health care by clinicians as well as administrative leaders [42]. If PRO systems are to be a central part of patient care, they must allow the majority of the patients to be included. Few departments will invest time in systems that just create an additional pathway for patients. The goal should be to include the vast majority of the outpatients, acknowledging that a small proportion will always need individualized services and care.

### Modes of PRO Collection

The term TeleHealth emphasizes a physical distance between patient and clinician, while the term ePRO just signals that the mode includes some electronic device in the hospital or at home. We recommend the use of the term TelePRO when PRO is collected at a distance, regardless of which mode is used.

While nearly all patients have access to the internet, about 50% of patients are not capable or willing to fill out TelePRO questionnaires on the Web when they are asked to do so. This is a consistent finding in all published randomized studies in patient populations [18-20]. Consequently, ePRO systems alone cannot be used for TelePRO when high participation rates are wanted. This problem is frequently ignored, and reports of ePRO data collection should at least document and report the actual proportion of patients included [23]. A mixed-mode approach may overcome this limitation and reach a Web-share of 55% to 60% without jeopardizing the response rate. When all functions, including printing and scanning of paper forms, are fully automated, the marginal cost of using paper forms as a fall-back method is limited compared with the advantage of response rates of up to 93%. Finally, ePRO solutions also require resources [42]. A large number of the support inquiries concerning projects implemented within WestChronic are about the patients’ problems with the Internet, browser, or email account.

ePRO questionnaires are generally supposed to produce data that are equivalent to the data produced from the paper version if modifications of content and format are minimal [17,25]. We use the same software to generate both versions, which means the two modes as close to each other as possible.

### Content of Questionnaires for Clinical Use

Validity and reliability are cornerstones in clinical epidemiology, and other key attributes, include interpretability of scores and acceptable burdens for both patients and clinicians [24]. However, it all depends on the type of inference to be drawn from the PRO measure. To analyze group-level PRO, we should be concerned about biased estimates, whereas when PRO is used for screening, we should focus on sensitivity and predictive values. Each instrument must be evaluated for the exact clinical application. In the DanPROM project, such questionnaires were used for assessment of need for control after hip or knee replacement [36,37]. However, they were not able to discriminate between symptoms from the contralateral knee or hip. Bilateral symptoms are common, and high symptom scores may just mean that the patient is waiting for surgery of the other hip or knee. This is an example of an instrument that may be valid for some purposes, but may turn out to be less useful at an individual level.

Most clinical decisions are of qualitative nature and based on a number of inputs. When PRO measures are used for clinical decision support, they are also most often used together with clinical information (eg, in an EHR system). When PRO measures are used to decide whether a patient should be seen or not, the covariates for this dichotomous decision may not be operational in epidemiological terms, and the actual decisions not based on empirical evidence but on clinical experience and practice [24]. Group level PRO questionnaires, if available, may not be relevant or accepted by patients or the clinicians, who have to rely on them when evaluating the PRO overview (optional element 1). This form of validity, face validity, is fundamental as seen from the clinician’s point of view and should be ensured during the implementation process for each new patient group. While a number of recommendations can be considered for group level PRO [24,25], these are not applicable to all aspects and domains at the patient level. Furthermore, summary scores or empirically based cut-offs are often not clinically relevant or applicable. Because the clinician still has the full responsibility for the decision, we constructed the wording as close as possible to the actual wording of questions the clinician asks the patients in daily practice. In this way we ensured the face validity.

Clinicians may be reluctant to gather too much information because they have to respond and react to issues with regard to which they may feel they lack competence (eg, assessment of signs of depression). The development of guidelines on how to react has been proposed to comply with this problem [43].

### Patient Safety in PRO for Clinical Use

When PRO measures are used for clinical monitoring and decisions, it is considered a medical device and should comply with regulations with respect to documentation of safety [44]. Sensitivity of automatic procedures, for example cancelling of appointments, should be maximal, but often hardly possible to describe in relevant quantitative terms. Furthermore, there may be issues not included in the questionnaire that definitely necessitate a consultation. We recommend that PRO questionnaires for automated decisions should include items that give a possibility for the patient to overrule an automated decision. This is not only important for patient safety, but since the patient most likely can deduce which answers qualify for a desired algorithm-based decision, it is a prerequisite for getting trustworthy answers. It is also vital to ensure that nonresponding patients are not lost from clinical follow-up if they do not react to questionnaires or reminders. Such patients should be invited to a normal outpatient visit.

### Implementation in Clinical Practice

AmbuFlex (project types D+G) has been implemented in clinical practice for different groups of patients and for different purposes, and the Central Region of Denmark has recently decided to extend the use of AmbuFlex to three new diagnostic groups every year. The integration into the EHR system has increased the impact considerably. The data available so far suggest that PRO-based clinical systems implemented in close teamwork with involved clinicians may be a suitable instrument with respect to quality improvement and intelligent resource utilization. However, not all groups of outpatients are suitable for systems like AmbuFlex. Information obtained from the patient should be of major importance in the clinical assessment of the disease, if necessary with support from biochemical or other laboratory and imaging data, but a physical examination should not be central for evaluation of the clinical status. If PRO is used to avoid needless consultations, the variation in turn-around time for PRO collection should be considered. Special appointments reserved for PRO patients are recommended instead of cancellation of prescheduled appointments. In the implementation process for a new diagnostic group, involvement of the patients as well as support from frontline clinicians and administrative leaders are essential.

### Conclusions

Organizational research has introduced the concept of disruptive innovation and applied it to health care [42]. Incorporating PRO at the center of health care, not as an adjunct and added task to usual care, can be viewed as a disruptive innovation. This could occur if clinicians and administrative leaders take patient-centered care so seriously that PRO, not the patient visit, are the center of the model [42]. If this point is reached, overloaded outpatient clinics would potentially be able to skip large numbers of prescheduled routine review visits, which often occur despite the patient being well and no action being required, and instead direct resources toward the patients with real needs [45].

PRO have been collected and used for decades, but mostly as part of projects sharply confined in time and space. Based on our experiences, we can put forward the following suggestions to promote PRO collection as a permanent activity.

First, the focus should be on development of generic (not diagnosis-specific) models. Most long-term conditions have communalities that make the use of the same technology desirable [22].

Second, response rates are important for any purpose. If the target group is patients who show up at the outpatient clinic, a patient kiosk system at the hospital is a relatively simple solution. If the target group is all patients, a TelePRO solution is needed, and mixed-mode PRO collection should be considered to reach the majority of the patient group.

Finally, the PRO data should be relevant for several purposes. Data collected according to routine may be useful at a group level for assessment of hospital performance as well as clinical research. If PRO data can be used at both individual and group levels (eg, clinical research or quality improvement), collection of such data is more likely to be considered worthwhile, and thus to be implemented on a permanent basis.

## Multimedia Appendix 1

Automatic optimization of paper/ePRO modes in WestChronic, a mixed-mode PRO system.

## Abbreviations

ADHD: attention deficit hyperactivity disorder
CPR: central personal registry
EHR: electronic health record
ePRO: patient-reported outcome data electronically
N/A: not applicable
PRO: patient-reported outcome
RR1: minimal response rate TeleHealth: a physical distance between patient and clinician
TelePRO: patient-reported outcome data provided at a distance
