# Supplementary material for: Use of Patient-Reported Outcome (PRO) Measures at Group and Patient Levels: Experiences From the Generic Integrated PRO System, WestChronic
Source: Interact J Med Res. 2014 Feb 11;3(1):e5. doi: 10.2196/ijmr.2885 (PMC3936283; doi:10.2196/ijmr.2885)

Multimedia Appendix 1

Automatic Optimization of Paper/ePRO Modes in WestChronic, a Mixed-Mode PRO System.

The encouragement and reminder procedures are dynamic and fully automatically. The first questionnaire is a paper form sent by post in almost all implemented projects (figure below). In the last question in this questionnaire, we ask the patient to indicate whether s/he is willing to answer the next PRO measure by a Web-questionnaire. If so, at the time of the next scheduled PRO measure, a letter is mailed with instructions on how to answer the Web-questionnaire. In the Web-based questionnaire, the last question is replaced with a question on whether the patient is willing to be contacted by email at the time of the next scheduled questionnaire and if so, to indicate an email address. This mechanism ensures transfer of the patient to the cheapest possible method of data collection.

WestChronic projects may involve from 0 to 3 reminders. If the patient does not answer, a reminder is sent after, for example 14 days, depending on the protocol of the actual project. The mode for the reminder is the method to the left of the last used approach. If the patient was approached by email, the reminder is a postal reminder with instructions on Web-questionnaires. If the last approach was a posted letter, the reminder is a letter including a paper questionnaire. This mechanism ensures transfer of the patient to the cheapest mode with response.

Together the two mechanisms constitute a self-regulating system, which promotes a dynamically optimal equilibrium between low cost and high response rate. The proportions of Web-based answers are between 50% and 60% after the second round. More than 15% of patients change mode in each round. If an ePRO system is used, all nonrespondents to the method will be lost.

Automatic dynamic switching between modes

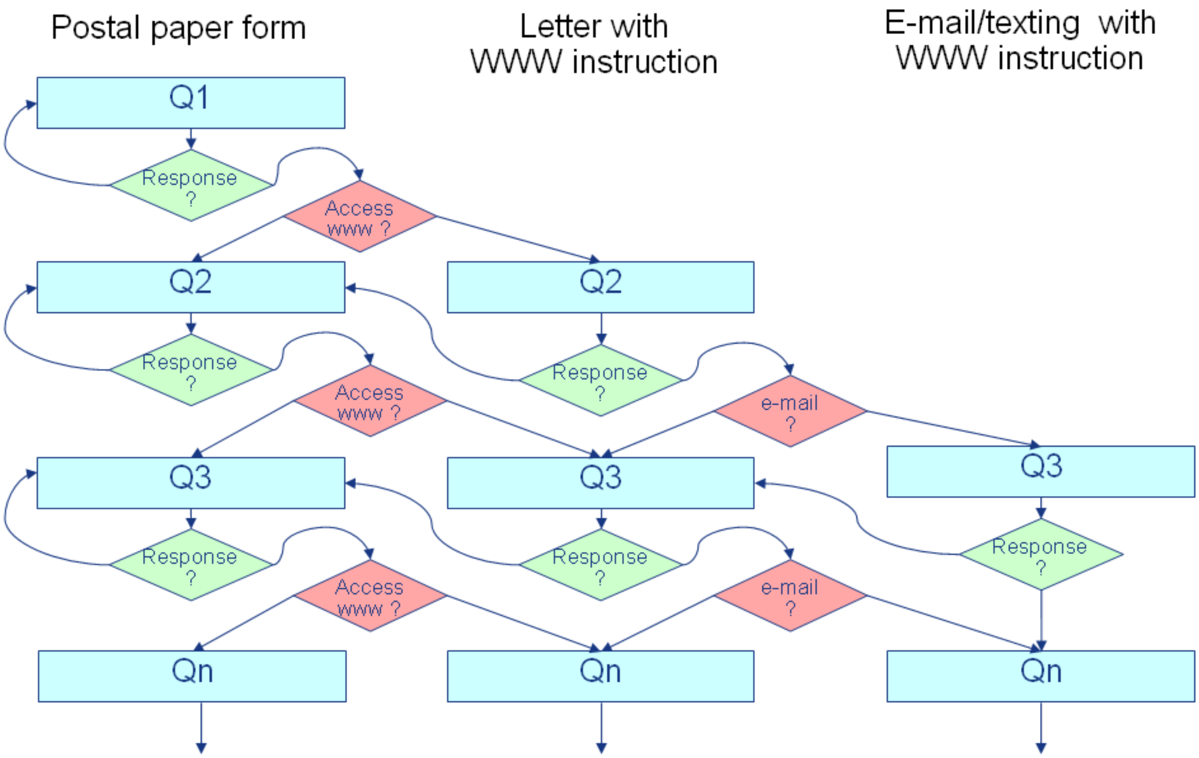

Supplement: Supplementary file 1 [file ijmr_v3i1e5_app1.pdf]
